# Continuity and changes in grandchild care and the risk of depression for Chinese grandparents: new evidence from CHARLS

**DOI:** 10.3389/fpubh.2023.1217998

**Published:** 2023-08-03

**Authors:** Yue Hong, Wei Xu

**Affiliations:** School of Sociology, Wuhan University, Wuhan, China

**Keywords:** grandchild care, depression, changes in caregiving intensity, rural–urban context, gender norms

## Abstract

**Objectives:**

Although studies have researched the mental effects of intergenerational care, little is known about the impact of transformations in caregiving intensity on depression. This study explores grand-parents’ depressive symptom outcomes in terms of changes over time in grandparental childcare, with considerations for subgroup differences.

**Method:**

Using data from the 2015–2018 China Health and Retirement Longitudinal Study on grandparents aged 45 and older, we adopted generalized estimating equations to estimate the effects of seven category changes [(1) continued to provide high-intensity or (2) low-intensity care at both waves; (3) never provided care; (4) started caregiving; (5) ended caregiving; (6) provided less intensive care; and (7) provided more intensive care] over time in grandparental childcare on depressive symptoms among 17,701 grandparents with at least one grandchild, as well as how the impact varies by gender and urban/rural areas.

**Results:**

Grandparents who decreased the intensity of care, stopped childcare, or offered continuous low-intensity care were associated with a lower level of depression compared with those providing no childcare. In addition, the benefit of continuous caregiving on mental health was especially noticeable in urban grandmothers.

**Conclusion:**

Providing continuous low-intensity, decreased-intensity grandparenting and the cessation of caregiving were associated with a decreased level of depression for Chinese grandparents; however, there were complex interactions at play. Policies aimed at supporting grandparenting should consider caregiving intensity transitions relevant to gender and urban/rural residence.

## 1. Introduction

In China, grandparents play increasingly active roles in providing care for their grandchildren (1). Over 50 percent of Chinese middle-aged and older grandparents participate in grandchild care (2). Strong cultural traditions of filial piety and the norm of intergenerational reciprocity promote Chinese grandparents to take on the responsibility of caring grandchildren (3). Especially in rural areas, large numbers of migrants have left their children in the care of grandparents at home (4). It has been reported that more than a quarter of children in rural China live solely with a grandparent (5). With increased life expectancy, more Chinese grandparents can care for their parents and grandchildren to alleviate the time and financial strain on young couples raising children (6). In this context, the question of whether long-term care activities promote or impair the mental health of older caregivers has attracted scholars’ attention.

The impacts of looking after children on grandparents’ mental health are controversial. On the one hand, caregiving activities can be a physical burden and limit grandparents’ time and opportunities to care for themselves (7) and assume the role of marriage (8), which makes it harder for caregivers to resist emotional stress. On the other hand, grandparents may feel higher life satisfaction, self-efficacy (9) and less depression (10) when increasing intergenerational contact by engaging in caring for grandchildren (11). Moreover, some findings have also demonstrated null effects between caregiving and caregivers’ depression symptoms (4).

After controlling for socioeconomic and demographic characteristics, a growing body of literature suggests that the impact of grandparenting on grandparents’ depression is contingent on the intensity of parenting and its change and continuity. Some articles highlighted the importance of grandparenting for a moderate level of engagement (12, 13). Only a few articles have deeply researched the effect of dynamic changes in grandparenting on depression. Ku et al. (14) indicated that grandparents who continued to provide care reported fewer depressive symptoms than non-caregivers. However, Di Gessa et al. (15) suggested that there was no significant link in European countries between the change in grandparental childcare and depression symptoms. Such an effect may not be fully detected if there are no measures of care intensity and no strong evidence from the Chinese cultural context.

There has been some literature about the relation between caring for grandchildren and grandparents’ depression symptoms. However, apart from using data from America or European countries (15–17), research on grandparenting in China adopted mostly cross-sectional data (18) or longitudinal samples in specific regions (19), which made it difficult to ascertain external validity and the extensibility of the sample. Moreover, previous studies have tended to focus on the health impacts of whether grandparents participate in grandparenting (19) or caregiving intensity (13). Research on how the duration of grandchild care influenced caregivers’ health mainly used simple classifications of grandchild care based on whether grandparents provided care (7, 14, 20), which considered little about transformations in caregiving intensity as well as subgroup variations in gender and urban/rural residence (13). Specifically, the variations in measures of care intensity may cause inconsistent analysis results. Some analyses have used the time of providing caregiving, such as weekly (21) and yearly hours (22), to differentiate levels of intensity. Besides, there were inconsistent measures of adopting continuous metrics or categories to be determined as appropriate, such as adopting a continuous variable of caregiving time (23) or providing less or more than 4 h in helping grandchildren per week (21). Other works in the literature employed a functional definition of caregiving status (24).

## 2. Literature review

### 2.1. Grandchild care and depressive symptoms

Caregiving can predict the changes in depression symptoms both positively and negatively. Role Accumulation Theory and Role Tension Theory are competing perspectives that have been widely used to study the impacts of grandparenting on mental health. Role Accumulation Theory postulates that multiple social roles are associated with higher life satisfaction and self-efficacy (9) when achieving social integration in different areas (25). As individuals age, retire, and become less socially integrated, the need to strengthen social roles may be greater than ever (26). As a kind of social activity, caring for grandchildren meets intergenerational emotional needs (10) and promotes social integration (27) for grandparents through meaningful engagement. Role Tension Theory, in contrast, suggests that individuals may face role conflicts and role stress when requested to perform specific obligations of different roles (28). Providing childcare may limit grandparents’ time and opportunities to care for themselves (29) and assume the role of marriage (8), making them more vulnerable to emotional stress. Furthermore, older people frequently feel unvalued and disrespected as a result of generational conflicts over child-rearing understanding, which is harmful to psychological health (30).

### 2.2. Rural–urban context and gender norms in China

Rural–urban residence and gender roles shape intergenerational care and depressive symptoms for Chinese grandparents (13). The traditional family division of labour has shaped varied responsibilities between grandmothers and grandfathers. Women are expected to take on more intensive duties such as feeding and cooking, while men usually play a companion role as playmates or helpers (31). In this way, engaging in the care of grandchildren is regarded as a deviation from the norm for men (31). As shown in a previous study, compared with grandmothers, grandfathers suffered from a greater risk of depression when continuously providing high-intensity care (22). However, because women are the main caregivers, some findings showed that the positive mental health outcomes of caregiving were reported significantly only for grandmothers, whereas this was previously thought to be the effect of the whole sample (21, 32). In addition, when considering the intersection of gender roles and the rural–urban context, it may turn out that only urban grandfathers who were financially independent and did not seek an intergenerational time-for-money exchange were associated with fewer depressive symptoms from grandchild care (31). The health effects of continuous caring on gender and urban/rural subgroups have yet to be fully studied.

The consequences of grandchild care are associated with the residence circumstances of the care. First, in recent decades, numerous rural workers have migrated to urban areas, leaving their young children to be cared for by grandparents. It results in rural grandparents having to do more extensive care duties and becoming acclimated to it (1). Second, rural state pension coverage is less extensive than in urban areas, implying that intergenerational care is more of a burden than a reward for rural grandparents (31). This means that rural grandparents have to shoulder the financial and physical burden of caring for grandchildren (33). Third, the one-child policy, which was enforced more rigorously in urban areas, leads to greater involvement and less burden for urban grandparents (31). Affluent urban grandparents tend to compete for opportunities to nurture and care for shrinking numbers of grandchildren to gain emotional rewards (34). Compared to their urban counterparts, rural grandparents are more dependent on their children’s financial support (35–37). In this way, rural grandparents may be more likely to consider grandchild care as a reciprocal form of intergenerational reward rather than an emotionally rewarding activity (33). Forth, rural grandparents could be more likely to become the ‘sandwich’ generation at a young age and face conflicts between providing care for their parents and grandchildren (38), which may make intergenerational care particularly stressful.

### 2.3. The present study

Our research contributes to the literature in three ways. First, we used national panel data from China to investigate the relationship between stability and changes in grandparental childcare and depression, expanding on previous studies that were either cross-sectional or had specific region observation windows. Considering that little attention has been paid to the duration and changes of grandparenting on mental health, we classified it based on the intensity of caregiving rather than simple engagement. Second, we adopted generalized estimating equations to estimate the effects of caregiving transitions on depressive symptoms to handle time-dependent autocorrelated data (39). Finally, we performed an exploratory investigation to explore how the relationships between the changes in grandparenting intensity and depression vary between different subgroups of location and gender, while prior studies mostly focused on how the relations differ across gender (40). Based on current evidence, we expect that changes in grandparenting intensity is associated with depression symptoms in later life (Hypothesis 1). Furthermore, the effects of grandparenting change on depression operate differently by gender and urban/rural areas (Hypothesis 2).

## 3. Method

### 3.1. Data and sample

The data came from China Health and Retirement Longitudinal Surveys (CHARLS) hosted by the National Development Research Institute of Peking University. CHARLS adopted probability proportional to size sampling to collect a nationally representative sample of Chinese residents aged 45 and above, which included 450 villages in 150 counties and districts throughout China. We obtained national tracking survey data in 2015 and 2018 from the official website.^1^ Our analysis was limited to the respondents with follow-up information (*n* = 36,274) above 45 (*n* = 34,530) who reported having at least one grandchild under 16 at baseline (*n* = 25,186) and provided complete answers to the depression level (*n* = 21,434), the independent variable (*n* = 18,835), demographics and family characteristics (*n* = 17,701) in both waves (see Supplementary Figure S1). We compared the differences in socioeconomic characteristics between excluded and retained samples (see Supplementary Table S1), and the retained participants were younger, more males, more rural residences, more at married or cohabiting status, more at fair or good health status, at higher level of individual expenditure, and had higher levels of education, which also showed up in other similar studies (39, 41). Our samples were not significantly different in depressive symptoms when compared with the excluded samples, but it may be necessary to be careful to generalize our results.

### 3.2. Measures

The dependent variable was the level of depression. According to the epidemiology research center Depression Scale (CES-D), respondents were asked to assess their psychological and emotional states within a week. The scale included ten subitems. The 4-point responses were rescaled from little or no (0) to most of the time (3). The total score was between 0 and 30. The higher the score, the more serious the depression. Depression levels were log-transformed to fit a normal distribution of the variables and ranged from 0 to 3.434 (Cronbach *α* = 0.77).

We constructed annual hours of grandparenting to measure the intensity of care and built the classification of changes on that basis. In two waves, respondents with at least one grandchild were asked if they had cared for them in the previous 12 months. If they did, they were asked how many weeks and, on average, how many hours they would have cared for their grandchildren per week in the last year. Using this information, we distinguished between three types of grandparent care: high-intensity care (i.e., those who cared for grandchildren at least 40 h per week on average or for over 2080 h in the last year), low-intensity care (i.e., those who cared for grandchildren for less than 40 h per week on average or under 2080 h last year), and noncaregivers (i.e., those who did not care for grandchildren). We chose 40 h per week for intensive grandparent care because according to the Labor Law of China and previous studies, 40 h per week (five-day working week and no more than 8 h a day) is equivalent to having a full-time job (40, 42). Then, we created a 7-category stability showing the changes in the provision of grandchild care in two waves. We distinguished those who (1) continued to provide high-intensity or (2) low-intensity care at both waves; (3) never provided care; (4) started caregiving (did not care at Wave 1 but provided high-intensity or low-intensity care at Wave 2); (5) ended caregiving (only providing high-intensity or low-intensity care at Wave 1); (6) provided less intensive care (from high-intensity care at Wave 1 to low-intensity care at Wave 2); and (7) provided more intensive care (from low-intensity care at Wave 1 to high-intensity care at Wave 2).

The control variables at baseline included gender (female = 0); location (rural = 0); education level (illiterate = 0; primary school and below = 1; middle school graduation = 2; high school graduation = 3; college and above = 4); marital status (separation, divorce, widowhood, never married = 0; married, cohabiting = 1); self-rated health status (poor = 0; fair =1; good =2); age; whether to live with children (yes = 1); number of grandchildren under the age of 16; number of surviving children; and *per capita* household expenditure.

### 3.3. Data analysis

We compared the differences of depression levels and other baseline characteristics across four subgroups of rural grandmothers, rural grandfathers, urban grandmothers, and urban grandfathers (see Table 1) and seven category changes over time in grandparental childcare (see Supplementary Table S2), including the chi-square test and variance (ANOVA) for categorical variables and normally distributed continuous variables. Then, we set the models of generalized estimating equations (GEE) to evaluate the effects of changes in caregiving intensity on the level of depressive symptoms, which is flexible to analyse correlated data from the same subjects over time (43) and control the confounding variables that change over time, such as self-rated health status (44). Model 1 was specified to evaluate the effect of the caregiving transition on the dependent variable after taking into account a range of baseline covariates. To test Hypothesis 2 regarding whether the effect of transition of caregiving on depressive symptoms differed in gender and location groups, we divided subgroups in model 2, model 3, model 4 and model 5. Instead of cross-terms for care intensity with gender and urban/rural residence, we operated models by groups to better understand the internal characteristics of different samples.

**Table 1:** Comparison between subgroups at baseline.

	(1)	(2)	(3)	(4)	(5)	F/χ2
	Mean (SD)/*N* (%)	Mean (SD)/*N* (%)	Mean (SD)/*N* (%)	Mean (SD)/*N* (%)	Mean (SD)/*N* (%)	Significance
Depression	1.903 (0.857)	2.106 (0.814)	1.807 (0.835)	1.845 (0.861)	1.573 (0.899)	141.47***
Changes of caregiving intensity						91.143***
No childcare at either wave	3,056 (33.44)	1,248 (34.46)	1,204 (35.94)	305 (27.31)	299 (28.50)	
High-intensity childcare at both waves	985 (10.78)	408 (11.26)	332 (9.91)	126 (11.28)	119 (11.34)	
Low-intensity childcare at both waves	767 (8.39)	272 (7.51)	254 (7.58)	123 (11.01)	118 (11.25)	
Starting childcare at Wave 2	1,372 (15.01)	527 (14.55)	523 (15.61)	169 (15.13)	153 (14.59)	
Stopped childcare at Wave 2	1,539 (16.84)	631 (17.42)	574 (17.13)	171 (15.31)	163 (15.54)	
High-intensity → Low-intensity childcare	785 (8.59)	295 (8.14)	256 (7.64)	128 (11.46)	106 (10.10)	
Low-intensity → High-intensity childcare	634 (6.94)	241 (6.65)	207 (6.18)	95 (8.50)	91 (8.67)	
Male	4,399 (48.14)	0 (0.00)	3,350 (100.00)	0 (0.00)	1,049 (100.00)	9138.000***
Living in cities	2,166 (23.70)	0 (0.00)	0 (0.00)	1,117 (100.00)	1,049 (100.00)	9138.000***
Education						1728.087***
Illiterate	2,181 (23.87)	1,521 (41.99)	399 (11.91)	210 (18.80)	51 (4.86)	
Primary school and below	4,128 (45.17)	1,562 (43.13)	1728 (51.58)	443 (39.66)	395 (37.65)	
Middle school	1905 (20.85)	437 (12.07)	856 (25.55)	282 (25.25)	330 (31.46)	
High school	825 (9.03)	98 (2.71)	347 (10.36)	160 (14.32)	220 (20.97)	
College and above	99 (1.08)	4 (0.11)	20 (0.60)	22 (1.97)	53 (5.05)	
Married or cohabiting	8,250 (90.28)	3,187 (87.99)	3,120 (93.13)	956 (85.59)	987 (94.09)	98.159***
Self-assessed health status						133.870***
Poor	2,279 (24.94)	1,101 (30.40)	768 (22.93)	242 (21.67)	168 (16.02)	
Fair	4,744 (51.92)	1805 (49.83)	1748 (52.18)	618 (55.33)	573 (54.62)	
Good	2,115 (23.15)	716 (19.77)	834 (24.90)	257 (23.01)	308 (29.36)	
Age in 2015						68.139***
45–60	4,240 (46.40)	1832 (50.58)	1,459 (43.55)	514 (46.02)	435 (41.47)	
60–70	3,638 (39.81)	1,343 (37.08)	1,369 (40.87)	473 (42.35)	453 (43.18)	
70–80	1,132 (12.39)	404 (11.15)	454 (13.55)	125 (11.19)	149 (14.20)	
above 80	128 (1.40)	43 (1.19)	68 (2.03)	5 (0.45)	12 (1.14)	
Living with their children	3,349 (36.65)	1,319 (36.42)	1,173 (35.01)	444 (39.75)	413 (39.37)	11.909**
Number of grandchildren	2.592 (1.803)	2.736 (1.84)	2.777 (1.909)	2.072 (1.44)	2.056 (1.424)	83.52***
Number of children	2.76 (1.304)	2.9 (1.315)	2.855 (1.31)	2.399 (1.244)	2.361 (1.148)	83.22***
*Per capita* household expenditure	8.85 (1.081)	8.728 (1.084)	8.74 (1.054)	9.181 (1.035)	9.267 (1.032)	310.969***
1st quartile	2004 (21.93)	926 (25.57)	815 (24.33)	142 (12.71)	121 (11.53)	
2nd quartile	2,378 (26.02)	967 (26.70)	910 (27.16)	270 (24.17)	231 (22.02)	
3rd quartile	2,455 (26.87)	954 (26.34)	912 (27.22)	304 (27.22)	285 (27.17)	
4th quartile	2,301 (25.18)	775 (21.40)	713 (21.28)	401 (35.90)	412 (39.28)	
Number of observations	9,138	3,622	3,350	1,117	1,049	

**p < 0.01, ***p < 0.001. Model (1) to (5) describe samples of all samples, rural grandmothers, rural grandfathers, urban grandmothers, and urban grandfathers in turn.

## 4. Results

### 4.1. Descriptive statistics

Table 1 suggests descriptive information of the sample at baseline. According to the stability and change in grandchild care, only 33.44% of grandparents provided no care at either wave. Close to one-fifth of grandparents in Wave 2 continued to provide the same level of intergenerational care reported in Wave 1: 10.78% continued to provide high-intensity care, and 8.39% continued to provide low-intensity care in Wave 2. More than 20% of grandparents increased their level of grandparenting: 15.01% provided no care at Wave 1 and any care for grandchildren in Wave 2; 6.94% provided low-intensity care at Wave 1 and high-intensity care at Wave 2. More than 25% of grandparents decreased the intensity of intergenerational care: 16.84% cared for grandchildren at Wave 1 and ended up at Wave 2; 8.59% provided high-intensity care at Wave 1 and low-intensity care at Wave 2.

There were significantly differences among four groups. Rural grandmothers had the highest scores for depressive symptoms, and urban grandfathers scored the lowest. Rural grandparents had a higher proportion of noncaregivers than urban samples, while urban grandparents had a higher proportion of continuous low-intensity and decreased-intensity care. In total, urban grandfathers were the most educated, the least single, had the best self-assessed health status, had the least number of grandchildren, and had the highest level of individual household expenditure. However, rural grandmothers seemed to be the most disadvantaged in these socio-economic indicators.

### 4.2. Changes in caregiving intensity and depressive symptoms

The results of the longitudinal impact of grandparenting on depression are presented in Table 2. After controlling for sociodemographic characteristics and family information variables, stability and changes in grandparental childcare were associated with caregivers’ depressive symptoms. Hypothesis 1 was supported. Grandparents who provided high-intensity care in Wave 1 and low-intensity care in Wave 2, who stopped childcare at Wave 2, and who offered low-intensity care in 2 waves were associated with a lower level of depression compared with those providing no childcare. Associations with other covariates were broadly similar to previous studies. Males, living in urban areas, higher-level education, being married or cohabiting, better self-assessed health status, older age, less children, and higher-level of household individual expenditure were associated with decreased depression.

**Table 2:** GEE regression of caregiving changes on grandparents’ depression by subgroups.

	Model 1	Model 2	Model 3	Model 4	Model 5
	Estimate (SE)	Estimate (SE)	Estimate (SE)	Estimate (SE)	Estimate (SE)
Intercept	2.770*** (0.034)	2.800*** (0.048)	2.575*** (0.064)	2.800*** (0.103)	2.264*** (0.156)
Changes of caregiving intensity (ref: no childcare at either wave)					
High-intensity childcare at both waves	−0.002 (0.024)	0.077* (0.035)	−0.043 (0.042)	−0.136* (0.068)	0.017 (0.074)
Low-intensity childcare at both waves	−0.048† (0.027)	−0.047 (0.042)	−0.034 (0.046)	−0.155* (0.072)	0.083 (0.075)
Starting childcare at Wave 2	−0.024 (0.021)	−0.042 (0.032)	−0.027 (0.034)	−0.038 (0.064)	0.068 (0.066)
Stopped childcare at Wave 2	−0.052** (0.020)	−0.026 (0.031)	−0.105** (0.032)	−0.065 (0.060)	0.042 (0.065)
High-intensity childcare → Low-intensity childcare	−0.067* (0.026)	−0.101* (0.042)	−0.046 (0.046)	−0.104 (0.067)	0.029 (0.079)
Low-intensity childcare → High-intensity childcare	−0.036 (0.028)	0.025 (0.042)	−0.093† (0.049)	−0.124† (0.076)	0.009 (0.086)
Gender (male)	−0.187*** (0.015)				
Location (city)	−0.095*** (0.017)				
Education (ref: illiterate)					
Primary school and below	−0.034* (0.017)	−0.027 (0.022)	−0.020 (0.036)	−0.025 (0.051)	−0.154 (0.101)
Middle school	−0.146*** (0.022)	−0.174*** (0.035)	−0.125** (0.040)	−0.136* (0.060)	−0.187† (0.103)
High school	−0.242*** (0.028)	−0.268*** (0.068)	−0.215*** (0.048)	−0.204** (0.069)	−0.322** (0.108)
College and above	−0.302*** (0.075)	0.019 (0.044)	−0.392* (0.199)	−0.119 (0.143)	−0.409** (0.138)
Marital status (married or cohabiting)	−0.170*** (0.021)	−0.214*** (0.029)	−0.167*** (0.042)	−0.131* (0.055)	0.021 (0.087)
Self-assessed health status (ref: poor)					
Fair	−0.456*** (0.014)	−0.431*** (0.020)	−0.467*** (0.024)	−0.508*** (0.039)	−0.502*** (0.051)
Good	−0.817*** (0.018)	−0.771*** (0.029)	−0.782*** (0.030)	−0.969*** (0.052)	−0.950*** (0.059)
Age in 2015 (ref: 45–60)					
60–70	−0.049*** (0.015)	−0.024 (0.022)	−0.020 (0.025)	−0.157*** (0.040)	−0.083† (0.046)
70–80	−0.107*** (0.022)	−0.088* (0.036)	−0.078* (0.036)	−0.222*** (0.067)	−0.129* (0.064)
Above 80	−0.199*** (0.050)	−0.121 (0.077)	−0.209** (0.073)	−0.050 (0.157)	−0.350* (0.152)
Living with their children	−0.018 (0.013)	−0.032 (0.021)	−0.017 (0.022)	−0.033 (0.038)	0.050 (0.042)
Number of grandchildren	0.004 (0.004)	0.007 (0.006)	0.001 (0.006)	0.022** (0.008)	−0.017 (0.014)
Number of children	0.023*** (0.006)	0.003 (0.010)	0.025* (0.010)	0.036* (0.017)	0.084*** (0.022)
*Per capita* household expenditure (ref: 1st quartile)					
2nd quartile	−0.069*** (0.016)	−0.048* (0.023)	−0.103*** (0.026)	−0.102† (0.054)	−0.032 (0.065)
3rd quartile	−0.103*** (0.016)	−0.073** (0.025)	−0.109*** (0.026)	−0.221*** (0.055)	−0.065 (0.062)
4th quartile	−0.106*** (0.019)	−0.069* (0.029)	−0.124*** (0.031)	−0.203*** (0.057)	−0.048 (0.065)
Number of observations	17,701	6,935	6,512	2,188	2066

^†^*p* < 0.1, *p < 0.05, **p < 0.01, ***p < 0.001. Model (1) to (5) uses samples of all samples, rural grandmothers, rural grandfathers, urban grandmothers and urban grandfathers in turn.

**Figure 1:**
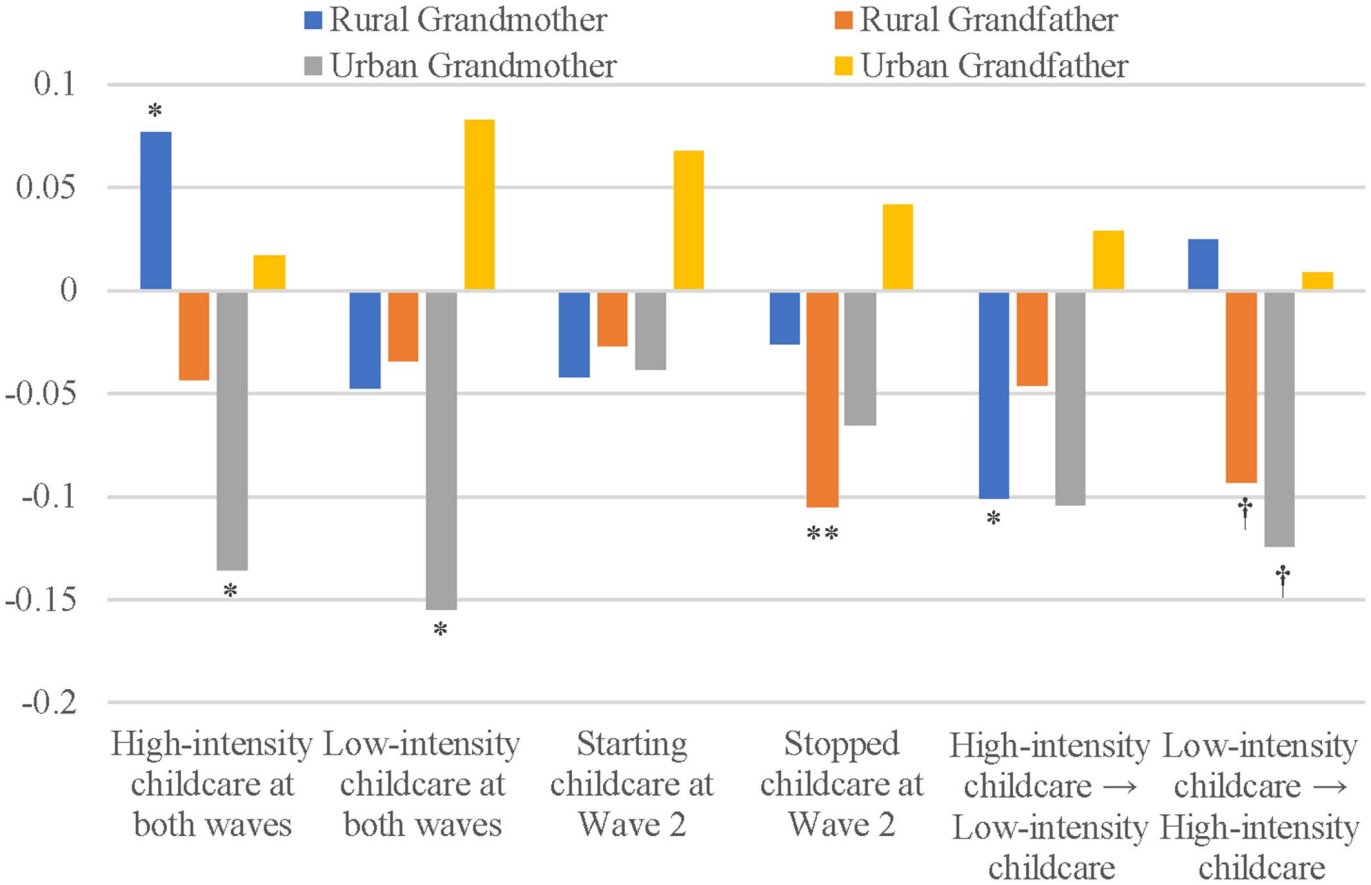
Effects of grandparenting intensity on health outcomes. The reference group of continuity and change in childcare is no childcare at either wave. Significance is based on the following values: ^†^*p* < 0.1, *p < 0.05, **p < 0.01. Results are based on models control variables given in Table 2.

The mental impact of change in grandparenting intensity revealed urban–rural and gender heterogeneity when respondents were divided into four groups. Hypothesis 2 was verified. Model 2 suggested that, compared with rural grandmothers who never participated in grandparenting, those who continued to provide high-intensity childcare were associated with an increased level of depression, and those who decreased the intensity of childcare were associated with a decreased level of depression. Model 3 showed that rural grandfathers who stopped childcare, and those who increased the intensity of childcare were associated with decreased depression when compared with noncaregivers. Model 4 suggested that urban grandmothers who provided continuous high-intensity or low-intensity childcare and those who increased the intensity of childcare were associated with decreased depression when compared with noncaregivers. Model 5 pointed out that the changes in childcare intensity were not significantly associated with urban grandfathers’ depressive symptoms. Figure 1 more clearly displayed differences across groups.

### 4.3. Supplementary analysis

We examined the effects of 16 classification changes in grandparenting intensity based on distinguishment between four types of grandparent care: high-intensity care (i.e., over 40 h per week on average), moderate-intensity care (i.e., more than 10 h but less than 40 h per week on average), low-intensity care (i.e., under 10 h per week on average) and noncaregivers (13). The results (see Supplementary Table S3) indicated that, compared with noncaregivers, grandparents who provided continuous moderate-intensity caregiving, decreased the levels of grandparenting from high intensity to lower intensity, stopped caregiving in Wave 2 from moderate-intensity or low-intensity care in Wave 1, and started caregiving at a low intensity in Wave 2, were associated with decreased levels of depressive symptoms. The results were roughly the same but more detailed than the results above. In addition, providing continuous low-intensity childcare and starting caregiving at a high intensity in Wave 2 were associated with increased depressive symptoms among urban grandfathers. More details were given in Supplementary Table S3.

## 5. Discussion

As Chinese grandparents play an increasingly important role in intergenerational care, the impact of care activities on old caregivers’ mental health has attracted scholars’ attention. Caregiving can predict the change in depressive symptoms in both positive and negative ways (12), while there are few studies on how the changes in caregiving intensity affects depression symptoms. The study provided longitudinal evidence on the stability of grandparental care’s effects, which further expanded the literature on how continuous grandparenting and its intersection of rural–urban and gender contexts impact mental health. Using the GEE model with longitudinal data from China, our findings suggested that the association between the changes in grandparenting intensity was not a simple one and varied in different groups. It appeared that stopping or decreasing the intensity of childcare was beneficial to grandparents’ mental health. Residence and gender can exert different influences on caregivers’ depression levels.

First, we confirmed that caregivers who continued caregiving could experience a lower level of depressive symptoms than their noncaregiving counterparts. This finding was similar to previous research (11, 14, 41, 45). And it is also in line with the Role Accumulation Theory which assumes that participating in caregiving activities may compensate grandparents for the loss of their former roles by injecting meaning into their lives (33). In particular, Chinese grandparents regard caring grandchildren as a productive role in Confucian filial piety culture (19). Based on research on the impacts of the continuity of caregiving, our study further provided strong evidence of the association between the changes in caregiving and depression among Chinese grandparents. The results emphasized the benefit of continuous moderate intensity care, decreased intensity of caregiving, and quitting intergenerational care. The advantage of moderate-intensity care has been pointed out before, while higher-intensity engagement could compromise health (12). Moreover, our findings were similar to the claim that the deterioration of grandparents’ health may contribute to the cessation of intergenerational care while stopping grandparenting provided an opportunity for grandparents to recover (20). However, some researchers supported that grandparents with cessation of care had a higher risk of depressive symptoms over time, and the strong loss of self-efficacy, as well as the social and financial isolations, may account for the results (45, 46). It appeared that these articles used data from other countries in the early 20th century, while our findings emphasized that in recent China there seemed to be no extra mental benefit of long-term intensive care for grandparents. Against the background of modernity, Chinese young grandparents, on the one hand, maintain a certain degree of emotional need in intergenerational interaction and, on the other hand, gradually acquire the characteristics of independence (47). The youngest grandparents (i.e., from 45 to 69 years old) received the most benefits of stopping and reducing the intensity of care, while the older grandparents did not (see Supplementary Table S4), which supported our interpretation to some extent. Furthermore, living with children was not found to be significantly associated with depressive symptoms in Chinese grandparents in the longitudinal study. It could be because older adults who did not live with children were more likely to receive financial assistance from children who could migrate and become better off, thereby preventing psychological damage for the old adults (36, 48). In addition, avoiding greater ambivalence toward family members also encourages parents to live apart from their children (49).

Second, our analyses of the conditional effects of care intensity changing by gender pointed out the compounding impact of gender norms and role theory on the links between caregiving and depressive symptoms. There were both similarities and heterogeneity in the impacts between rural grandfathers and rural grandmothers. On the one hand, reducing the intensity of care has a psychological bonus for rural grandmothers. It supported the Role Accumulation Theory that grandparents gained emotional fulfillment from the long-term family role, but the positive psychological effects became significant only if the role pressure was taken off, which suggested the applicability of Role Tension Theory to some extent. Similarly, rural grandfathers benefited from the cessation of caring. It showed the positive interactive effects of conforming to gender norms and taking on a caring role. On the other hand, increasing the intensity of caring for grandchildren could be beneficial for rural grandfathers, while rural grandmothers suffered from continuous high-intensity childcare. This finding was consistent with previous research showing that grandmothers may suffer more from the impact of role strain, such as being involved in high-intensity grandparenting (40). Caregiving activities seemed to be responsible and psychologically stressful for women, while they could be optional and joyful for men (31, 50). However, the impact of gender differences changed when it was estimated in urban society. Only urban grandmothers benefited from continuous caregiving, while urban grandfathers did not. It seems that the health benefit of providing sustained high-intensity care was especially noticeable among urban grandmothers. Caring was not associated with positive mental health outcomes for grandfathers in urban areas with strongly differentiated gender roles (21).

Third, findings on urban versus rural subgroups pointed out that the impacts of grandparenting on depressive symptoms were contingent on differential contexts. Compared with urban grandmothers acquired the psychological benefits from continuous caregiving at low intensity, increased intensity, and even high intensity, rural grandmothers only benefited from decreasing the intensity of grandparenting and suffered from continuous high-intensity care. These findings are consistent with previous research that urban grandparents tended to benefit more from intergenerational care than rural samples (31, 33). Because of less change in traditional norms and numerous left-behind children whose parents migrated for work in rural areas (51), compared with their urban peers, rural grandparents were more likely to take on intensive care and cope with psychological strains (2, 31). In addition, the lack of sufficient economic support may jeopardize the mental health of grandparents living in rural areas, while the availability of pensions and more financial resources helped to ease the burden of intensive caregiving for urban grandparents (33). Compared with rural grandfathers, involvement in grandparenting could not bring sufficient mental benefit that offset the negative impacts of deviation from gender norms for urban grandfathers, which revealed that there was no association between caring and depressive symptoms.

### 5.1. Limitations

There are some limitations in this study. First, specific contents of grandparenting activities, grandchild characteristics such as age or health, whether individuals care grandchildren collectively with their spouses, and the reasons for changes in caregiving, which may be associated with caregivers’ actual or perceived intensity of care activities but cannot be measured solely by caregiving time, were not available in the data. Thus, the impacts of intergenerational care could not be more accurate, and there may be some potential confounders that have not been controlled. Second, our study excluded participants who did not provide complete independent variables, dependent variables, and covariates, which may cause selection bias. Although there were no significant differences in depressive symptoms between the excluded samples and the included samples, the participants in our study had a higher likelihood of providing continuous caregiving and more favourable socioeconomic characteristics, which might be associated with a lower level of depression. This finding suggested that grandparents who were more capable of providing care might be oversampled, and favourable mental outcomes should be treated with caution. Third, although we used longitudinal data, these findings may not be a strict casual but only a causal inference under the social science paradigm, because intergenerational care and depressive symptoms were observed simultaneously.

## 6. Conclusion

By using longitudinal and nationally representative data from China, our study pointed out the effect of variation and persistence of caregiving intensity on depressive symptoms of grandparents. The results suggested that continuous low-intensity caregiving, decreased levels of involvement, and the cessation of caregiving were associated with fewer depressive symptoms for Chinese grandparents. In addition, the study found the detrimental effects of persistent high-intensity grandparenting and a beneficial effect of reduced-intensity care for rural grandmothers, while urban grandmothers took the advantages of continuous low-intensity care, increased level of involvement in caregiving and continuous high-intensity care. It also turned out that rural grandfathers who stopped caregiving or provided decreased-intensity care were more likely to experience fewer depressive symptoms. On the one hand, we could advocate for older adults to assist in caring grandchildren at a moderate intensity, which is conducive to active aging. On the other hand, with widespread participation in intergenerational caregiving, the potential risk of long-term and high-intensity caregiving on the mental health of older Chinese grandparents should be considered. Rural grandmothers, in particular, need much more family support and even community-based interventions to help provide supplementary care for left-behind children in order to alleviate persistent stress. More research using longitudinal data on the health impacts of continuous and varied caregiving is needed. Moreover, when it is considered that China is experiencing the historical situation of drastic modernization transformation, studies and any new policy related to them need to be placed in the context of nations, culture, and socioeconomic characteristics.

## Data availability statement

Publicly available datasets were analyzed in this study. This data can be found here: the dataset is available from the CHARLS repository, http://charls.pku.edu.cn.

## Ethics statement

The studies involving human participants were reviewed and approved by Institutional Review Board at Peking University. Ethics approval no. IRB00001052-11015. The patients/participants provided their written informed consent to participate in this study.

## Author contributions

WX and YH designed the study. YH contributed to the collection of literature, data processing, and result analysis and drafted the manuscript. WX contributed to the review of the manuscript. All authors contributed to the article and approved the submitted version.

## Conflict of interest

The authors declare that the research was conducted in the absence of any commercial or financial relationships that could be construed as a potential conflict of interest.

## Publisher’s note

All claims expressed in this article are solely those of the authors and do not necessarily represent those of their affiliated organizations, or those of the publisher, the editors and the reviewers. Any product that may be evaluated in this article, or claim that may be made by its manufacturer, is not guaranteed or endorsed by the publisher.

## Footnotes

^1^http://CHARLS.pku.edu.cn
