# Supplementary material for: Continuity and changes in grandchild care and the risk of depression for Chinese grandparents: new evidence from CHARLS
Source: Front Public Health. 2023 Aug 3;11:1217998. doi: 10.3389/fpubh.2023.1217998 (PMC10435994; doi:10.3389/fpubh.2023.1217998)
Supplement: Supplementary file 1 [file Table_1.docx]

Supplementary Material

# Supplementary Tables

Figure S1. Sample screening process

Table S1. Differences in socio-economic characteristics between excluded and retained samples

|  | Retained group | | Excluded group | | Total | | F/χ2 |
| --- | --- | --- | --- | --- | --- | --- | --- |
|  | Mean (N) | SD (%) | Mean (N) | SD (%) | Mean (N) | SD (%) | Significance |
| Depression | 1.944 | 0.856 | 1.941 | 0.86 | 1.943 | 0.857 | 0.08 |
|  | 17701 | 100.00 | 5693 | 100.00 | 23394 | 100.00 |  |
| Changes of caregiving intensity |  |  |  |  |  |  | 214.838*** |
| No childcare at either wave | 5751 | 32.49 | 1919 | 43.50 | 7670 | 34.69 |  |
| High-intensity childcare at both waves | 1982 | 11.20 | 356 | 8.07 | 2338 | 10.57 |  |
| Low-intensity childcare at both waves | 1507 | 8.51 | 294 | 6.66 | 1801 | 8.14 |  |
| Starting childcare at Wave 2 | 2724 | 15.39 | 561 | 12.72 | 3285 | 14.86 |  |
| Stopped childcare at Wave 2 | 2946 | 16.64 | 735 | 16.66 | 3681 | 16.65 |  |
| High-intensity childcare → Low-intensity childcare | 1546 | 8.73 | 294 | 6.66 | 1840 | 8.32 |  |
| Low-intensity childcare → High-intensity childcare | 1245 | 7.03 | 253 | 5.73 | 1498 | 6.77 |  |
| Total | 17701 | 100.00 | 4412 | 100.00 | 22113 | 100.00 |  |
| Gender |  |  |  |  |  |  | 23.803*** |
| Female | 9123 | 51.54 | 5545 | 54.57 | 14668 | 52.65 |  |
| Male | 8578 | 48.46 | 4616 | 45.43 | 13194 | 47.35 |  |
| Total | 17701 | 100.00 | 10161 | 100.00 | 27862 | 100.00 |  |
| Location of residential address |  |  |  |  |  |  | 49.104*** |
| Rural | 13447 | 75.97 | 7320 | 72.17 | 20767 | 74.58 |  |
| City | 4254 | 24.03 | 2823 | 27.83 | 7077 | 25.42 |  |
| Total | 17701 | 100.00 | 10143 | 100.00 | 27844 | 100.00 |  |
| What's your highest level of education? |  |  |  |  |  |  | 197.922*** |
| Illiterate | 4078 | 23.04 | 2750 | 30.68 | 6828 | 25.61 |  |
| Primary school and below | 8041 | 45.43 | 3785 | 42.23 | 11826 | 44.35 |  |
| Middle school | 3746 | 21.16 | 1585 | 17.68 | 5331 | 19.99 |  |
| High school | 1650 | 9.32 | 728 | 8.12 | 2378 | 8.92 |  |
| College and above | 186 | 1.05 | 115 | 1.28 | 301 | 1.13 |  |
| Total | 17701 | 100.00 | 8963 | 100.00 | 26664 | 100.00 |  |
| Marital status |  |  |  |  |  |  | 242.921*** |
| Single | 1884 | 10.64 | 1742 | 17.18 | 3626 | 13.02 |  |
| Married or cohabiting | 15817 | 89.36 | 8400 | 82.82 | 24217 | 86.98 |  |
| Total | 17701 | 100.00 | 10142 | 100.00 | 27843 | 100.00 |  |
| Self-assessed health status |  |  |  |  |  |  | 73.198*** |
| Poor | 4558 | 25.75 | 2624 | 30.74 | 7182 | 27.37 |  |
| Fair | 9043 | 51.09 | 4023 | 47.13 | 13066 | 49.80 |  |
| Good | 4100 | 23.16 | 1889 | 22.13 | 5989 | 22.83 |  |
| Total | 17701 | 100.00 | 8536 | 100.00 | 26237 | 100.00 |  |
| Age in 2015 |  |  |  |  |  |  | 503.305*** |
| 45-60 | 7323 | 41.37 | 3728 | 37.57 | 11051 | 40.01 |  |
| 60-70 | 7451 | 42.09 | 3723 | 37.52 | 11174 | 40.45 |  |
| 70-80 | 2597 | 14.67 | 1821 | 18.35 | 4418 | 15.99 |  |
| above 80 | 330 | 1.86 | 650 | 6.55 | 980 | 3.55 |  |
| Total | 17701 | 100.00 | 9922 | 100.00 | 27623 | 100.00 |  |
| Per capita household expenditure |  |  |  |  |  |  | 160.885*** |
| 1st quartile | 4660 | 26.33 | 3163 | 33.56 | 7823 | 28.84 |  |
| 2nd quartile | 4759 | 26.89 | 2376 | 25.21 | 7135 | 26.30 |  |
| 3rd quartile | 4632 | 26.17 | 2155 | 22.87 | 6787 | 25.02 |  |
| 4th quartile | 3650 | 20.62 | 1730 | 18.36 | 5380 | 19.83 |  |
| Total | 17701 | 100.00 | 9424 | 100.00 | 27125 | 100.00 |  |
| Note: ***p < .001 |  |  |  |  |  |  |  |

Table S2. Comparison between intensity changes at baseline

|  | (1) | | (2) | | (3) | | (4) | | (5) | | (6) | | (7) | | F/χ2 |
| --- | --- | --- | --- | --- | --- | --- | --- | --- | --- | --- | --- | --- | --- | --- | --- |
|  | (N = 3056) | | (N = 985) | | (N = 767) | | (N = 1372) | | (N = 1539) | | (N = 785) | | (N = 634) | |  |
|  | M/N | SD/% | M/N | SD/% | M/N | SD/% | M/N | SD/% | M/N | SD/% | M/N | SD/% | M/N | SD/% |  |
| Depression | 1.967 | 0.858 | 1.879 | 0.889 | 1.792 | 0.866 | 1.888 | 0.833 | 1.900 | 0.837 | 1.852 | 0.861 | 1.867 | 0.863 | 5.85*** |
| Gender |  |  |  |  |  |  |  |  |  |  |  |  |  |  | 5.914 |
| Female | 1553 | 50.82 | 534 | 54.21 | 395 | 51.50 | 696 | 50.73 | 802 | 52.11 | 423 | 53.89 | 336 | 53.00 |  |
| Male | 1503 | 49.18 | 451 | 45.79 | 372 | 48.50 | 676 | 49.27 | 737 | 47.89 | 362 | 46.11 | 298 | 47.00 |  |
| Location of Residential Address | | | | | | | | | | | | | | | 82.984*** |
| Rural | 2452 | 80.24 | 740 | 75.13 | 526 | 68.58 | 1050 | 76.53 | 1205 | 78.30 | 551 | 70.19 | 448 | 70.66 |  |
| City | 604 | 19.76 | 245 | 24.87 | 241 | 31.42 | 322 | 23.47 | 334 | 21.70 | 234 | 29.81 | 186 | 29.34 |  |
| What's Your Highest Level of Education? | | | | | | | | | | | | | | | 154.558*** |
| Illiterate | 836 | 27.36 | 209 | 21.22 | 138 | 17.99 | 291 | 21.21 | 409 | 26.58 | 171 | 21.78 | 127 | 20.03 |  |
| Primary school and below | 1469 | 48.07 | 425 | 43.15 | 346 | 45.11 | 613 | 44.68 | 685 | 44.51 | 314 | 40.00 | 276 | 43.53 |  |
| Middle school | 521 | 17.05 | 241 | 24.47 | 184 | 23.99 | 313 | 22.81 | 289 | 18.78 | 193 | 24.59 | 164 | 25.87 |  |
| High school | 206 | 6.74 | 97 | 9.85 | 81 | 10.56 | 140 | 10.20 | 141 | 9.16 | 100 | 12.74 | 60 | 9.46 |  |
| College and above | 24 | 0.79 | 13 | 1.32 | 18 | 2.35 | 15 | 1.09 | 15 | 0.97 | 7 | 0.89 | 7 | 1.10 |  |
| Marital Status |  |  |  |  |  |  |  |  |  |  |  |  |  |  | 100.733*** |
| Single | 416 | 13.61 | 56 | 5.69 | 52 | 6.78 | 103 | 7.51 | 160 | 10.40 | 65 | 8.28 | 36 | 5.68 |  |
| Married or cohabiting | 2640 | 86.39 | 929 | 94.31 | 715 | 93.22 | 1269 | 92.49 | 1379 | 89.60 | 720 | 91.72 | 598 | 94.32 |  |
| Self-assessed Health Status |  |  |  |  |  |  |  |  |  |  |  |  |  |  | 49.077*** |
| Poor | 863 | 28.24 | 211 | 21.42 | 163 | 21.25 | 327 | 23.83 | 400 | 25.99 | 173 | 22.04 | 142 | 22.40 |  |
| Fair | 1504 | 49.21 | 519 | 52.69 | 418 | 54.50 | 715 | 52.11 | 801 | 52.05 | 455 | 57.96 | 332 | 52.37 |  |
| Good | 689 | 22.55 | 255 | 25.89 | 186 | 24.25 | 330 | 24.05 | 338 | 21.96 | 157 | 20.00 | 160 | 25.24 |  |
| Age in 2015 |  |  |  |  |  |  |  |  |  |  |  |  |  |  | 848.459*** |
| 45-60 | 1032 | 33.77 | 624 | 63.35 | 424 | 55.28 | 757 | 55.17 | 597 | 38.79 | 415 | 52.87 | 391 | 61.67 |  |
| 60-70 | 1249 | 40.87 | 307 | 31.17 | 300 | 39.11 | 518 | 37.76 | 716 | 46.52 | 333 | 42.42 | 215 | 33.91 |  |
| 70-80 | 671 | 21.96 | 53 | 5.38 | 42 | 5.48 | 96 | 7.00 | 210 | 13.65 | 33 | 4.20 | 27 | 4.26 |  |
| above 80 | 104 | 3.40 | 1 | 0.10 | 1 | 0.13 | 1 | 0.07 | 16 | 1.04 | 4 | 0.51 | 1 | 0.16 |  |
| Whether are grandparents living with their children? 239.279*** | | | | | | | | | | | | | | | |
| No | 2247 | 73.53 | 518 | 52.59 | 446 | 58.15 | 846 | 61.66 | 948 | 61.60 | 443 | 56.43 | 341 | 53.79 |  |
| Yes | 809 | 26.47 | 467 | 47.41 | 321 | 41.85 | 526 | 38.34 | 591 | 38.40 | 342 | 43.57 | 293 | 46.21 |  |
| Number of Grandchildren | 2.443 | 1.784 | 2.956 | 1.930 | 2.254 | 1.501 | 2.552 | 1.769 | 2.835 | 1.937 | 2.636 | 1.615 | 2.590 | 1.822 | 19.75*** |
| Number of Children | 3.147 | 1.428 | 2.512 | 1.158 | 2.283 | 1.044 | 2.652 | 1.153 | 2.836 | 1.334 | 2.327 | 1.077 | 2.450 | 1.089 | 96.42 *** |
| Per capita household expenditure　 56.819*** | | | | | | | | | | | | | | | |
| 1st quartile | 724 | 23.69 | 213 | 21.62 | 137 | 17.86 | 270 | 19.68 | 361 | 23.46 | 170 | 21.66 | 129 | 20.35 |  |
| 2nd quartile | 759 | 24.84 | 293 | 29.75 | 191 | 24.90 | 360 | 26.24 | 417 | 27.10 | 184 | 23.44 | 174 | 27.44 |  |
| 3rd quartile | 857 | 28.04 | 256 | 25.99 | 193 | 25.16 | 388 | 28.28 | 390 | 25.34 | 211 | 26.88 | 160 | 25.24 |  |
| 4th quartile | 716 | 23.43 | 223 | 22.64 | 246 | 32.07 | 354 | 25.80 | 371 | 24.11 | 220 | 28.03 | 171 | 26.97 |  |

Note: (1) = No childcare at either wave; (2) = High-intensity childcare at both waves; (3) = Low-intensity childcare at both waves; (4) = Starting childcare at Wave 2; (5) = Stopped childcare at Wave 2; (6) = High-intensity childcare → Low-intensity childcare; (7) = Low-intensity childcare → High-intensity childcare. ***p < .001.

Table S3. GEE regression of caregiving change (16 types) on grandparents' depression

|  | Total | Rural Grandmother | Rural Grandfather | Urban Grandmother | Urban Grandfather |
| --- | --- | --- | --- | --- | --- |
|  | (N = 17701) | (N = 6935) | (N = 6512) | (N = 2188) | (N = 2066) |
|  | Estimate | Estimate | Estimate | Estimate | Estimate |
|  | (SE) | (SE) | (SE) | (SE) | (SE) |
| Intercept | 2.772*** | 2.802*** | 2.575*** | 2.811*** | 2.285*** |
|  | -0.034 | -0.048 | -0.064 | -0.103 | -0.155 |
| Change of depression level (ref: no childcare at either wave) |  |  |  |  |  |
| High-intensity childcare at both waves | -0.002 | **0.079*** | -0.043 | **-0.136*** | 0.015 |
|  | -0.024 | -0.035 | -0.042 | -0.068 | -0.074 |
| Moderate-intensity childcare at both waves | **-0.071†** | -0.03 | -0.119 | -0.05 | -0.011 |
|  | -0.04 | -0.062 | -0.074 | -0.092 | -0.116 |
| Low-intensity childcare at both waves | 0.021 | **-0.147†** | 0.078 | 0.169 | **0.243†** |
|  | -0.052 | -0.083 | -0.079 | -0.186 | -0.128 |
| No childcare → High-intensity childcare | -0.001 | -0.024 | -0.026 | -0.077 | **0.227**** |
|  | -0.03 | -0.046 | -0.052 | -0.081 | -0.086 |
| No childcare → Moderate-intensity childcare | -0.017 | -0.031 | 0.011 | -0.006 | -0.063 |
|  | -0.032 | -0.051 | -0.05 | -0.098 | -0.091 |
| No childcare → Low-intensity childcare | **-0.062†** | -0.074 | -0.066 | -0.023 | -0.021 |
|  | -0.034 | -0.051 | -0.053 | -0.117 | -0.114 |
| High-intensity childcare → No childcare | -0.022 | 0.022 | **-0.093*** | -0.045 | 0.05 |
|  | -0.027 | -0.042 | -0.046 | -0.079 | -0.082 |
| Moderate-intensity childcare → No childcare | **-0.087**** | -0.039 | **-0.154**** | **-0.193*** | 0.106 |
|  | -0.032 | -0.05 | -0.048 | -0.098 | -0.114 |
| Low-intensity childcare → No childcare | **-0.060†** | -0.078 | -0.074 | 0.021 | -0.031 |
|  | -0.033 | -0.048 | -0.054 | -0.095 | -0.109 |
| High-intensity childcare → Moderate-intensity childcare | **-0.063*** | **-0.085†** | -0.04 | -0.1 | -0.009 |
|  | -0.03 | -0.05 | -0.054 | -0.073 | -0.086 |
| High-intensity childcare → Low-intensity childcare | **-0.074†** | **-0.128*** | -0.059 | -0.111 | 0.15 |
|  | -0.043 | -0.063 | -0.075 | -0.114 | -0.137 |
| Moderate-intensity childcare → Low-intensity childcare | -0.071 | 0.004 | -0.044 | **-0.273*** | -0.004 |
|  | -0.052 | -0.074 | -0.097 | -0.118 | -0.153 |
| Low-intensity childcare → High-intensity childcare | -0.023 | 0.03 | -0.037 | -0.169 | -0.011 |
|  | -0.042 | -0.065 | -0.072 | -0.112 | -0.115 |
| Low-intensity childcare → Moderate-intensity childcare | -0.056 | -0.015 | -0.014 | **-0.358**** | 0.149 |
|  | -0.052 | -0.091 | -0.091 | -0.118 | -0.113 |
| Moderate-intensity childcare → High-intensity childcare | -0.044 | 0.024 | **-0.132*** | -0.093 | 0.018 |
|  | -0.034 | -0.05 | -0.06 | -0.088 | -0.108 |
| Gender (male) | -0.187*** |  |  |  |  |
|  | -0.015 |  |  |  |  |
| Location (city) | -0.096*** |  |  |  |  |
|  | -0.017 |  |  |  |  |
| Education (ref: illiterate) |  |  |  |  |  |
| Primary school and below | -0.033† | -0.025 | -0.018 | -0.031 | -0.155 |
|  | -0.017 | -0.022 | -0.036 | -0.051 | -0.102 |
| Middle school | -0.146*** | -0.175*** | -0.122** | -0.140* | -0.192† |
|  | -0.022 | -0.035 | -0.04 | -0.06 | -0.103 |
| High school | -0.241*** | -0.266*** | -0.214*** | -0.212** | -0.320** |
|  | -0.029 | -0.068 | -0.048 | -0.068 | -0.108 |
| College and above | -0.302*** | 0.03 | -0.412* | -0.167 | -0.400** |
|  | -0.076 | -0.048 | -0.203 | -0.147 | -0.138 |
| Married or cohabit | -0.171*** | -0.214*** | -0.167*** | -0.133* | 0.011 |
|  | -0.021 | -0.029 | -0.042 | -0.054 | -0.085 |
| Self-assessed health status (ref: poor) |  |  |  |  |  |
| Fair | -0.456*** | -0.430*** | -0.467*** | -0.506*** | -0.501*** |
|  | -0.014 | -0.02 | -0.024 | -0.039 | -0.051 |
| Good | -0.817*** | -0.771*** | -0.783*** | -0.972*** | -0.951*** |
|  | -0.018 | -0.029 | -0.03 | -0.053 | -0.059 |
| Age in 2015(ref: 45-60) |  |  |  |  |  |
| 60-70 | -0.049*** | -0.024 | -0.02 | -0.163*** | -0.083† |
|  | -0.015 | -0.022 | -0.025 | -0.04 | -0.045 |
| 70-80 | -0.107*** | -0.089* | -0.079* | -0.224*** | -0.135* |
|  | -0.022 | -0.036 | -0.036 | -0.067 | -0.063 |
| above 80 | -0.196*** | -0.118 | -0.204** | -0.054 | -0.346* |
|  | -0.05 | -0.077 | -0.073 | -0.162 | -0.152 |
| Whether are grandparents living with their children or not? (yes) | -0.019 | -0.035† | -0.017 | -0.034 | 0.052 |
|  | -0.013 | -0.021 | -0.022 | -0.038 | -0.042 |
| Number of grandchildren | 0.004 | 0.006 | 0.001 | 0.022** | -0.018 |
|  | -0.004 | -0.006 | -0.006 | -0.008 | -0.014 |
| Number of children | 0.023*** | 0.003 | 0.024* | 0.037* | 0.081*** |
|  | -0.006 | -0.01 | -0.01 | -0.017 | -0.022 |
| Per capita household expenditure (ref: 1st quartile) |  |  |  |  |  |
| 2nd quartile | -0.069*** | -0.049* | -0.102*** | -0.107* | -0.036 |
|  | -0.016 | -0.023 | -0.026 | -0.054 | -0.064 |
| 3rd quartile | -0.103*** | -0.074** | -0.109*** | -0.227*** | -0.066 |
|  | -0.016 | -0.025 | -0.026 | -0.055 | -0.062 |
| 4th quartile | -0.107*** | -0.069* | -0.128*** | -0.212*** | -0.052 |
|  | -0.019 | -0.029 | -0.031 | -0.057 | -0.065 |

Note: † p < 0.1, * p<0.05, ** p<0.01, *** p<0.001.

Table S4. GEE regression of caregiving change on grandparents' depression by age groups

|  | 45-59 | 60-69 | 70-79 | Above 80 |
| --- | --- | --- | --- | --- |
|  | (N = 7323) | (N = 7451) | (N = 2597) | (N = 330) |
|  | Model 1 | Model 2 | Model 3 | Model 4 |
|  | Estimate | Estimate | Estimate | Estimate |
|  | (SE) | (SE) | (SE) | (SE) |
| Intercept | 2.891*** | 2.695*** | 2.699*** | 2.466*** |
|  | (0.058) | (0.046) | (0.070) | (0.169) |
| Changes of caregiving intensity (ref: no childcare at either wave) | | |  |  |
| High-intensity childcare at both waves | -0.026 | -0.010 | 0.106 | -0.022 |
|  | (0.034) | (0.039) | (0.068) | (0.227) |
| Low-intensity childcare at both waves | -0.037 | -0.054 | -0.127 | -0.182 |
|  | (0.039) | (0.039) | (0.092) | (0.514) |
| Starting childcare at Wave 2 | -0.026 | -0.038 | -0.007 | -0.221 |
|  | (0.031) | (0.031) | (0.059) | (0.193) |
| Stopped childcare at Wave 2 | -0.051 | **-0.088**** | 0.008 | -0.097 |
|  | (0.035) | (0.028) | (0.042) | (0.138) |
| High-intensity childcare → Low-intensity childcare | **-0.132***** | -0.052 | 0.092 | 0.356 |
|  | (0.040) | (0.036) | (0.086) | (0.217) |
| Low-intensity childcare → High-intensity childcare | **-0.084*** | 0.009 | -0.068 | **0.602***** |
|  | (0.039) | (0.043) | (0.102) | (0.142) |
| Gender (male) | -0.203*** | -0.178*** | -0.161*** | -0.361*** |
|  | (0.022) | (0.022) | (0.037) | (0.096) |
| Location (city) | -0.048† | -0.144*** | -0.092* | 0.067 |
|  | (0.025) | (0.026) | (0.045) | (0.124) |
| Education (ref: illiterate) |  |  |  |  |
| Primary school and below | -0.009 | -0.037 | -0.091* | 0.126 |
|  | (0.029) | (0.025) | (0.040) | (0.096) |
| Middle school | -0.121*** | -0.145*** | -0.174** | -0.160 |
|  | (0.032) | (0.034) | (0.056) | (0.167) |
| High school | -0.205*** | -0.251*** | -0.256** | -0.617* |
|  | (0.041) | (0.043) | (0.082) | (0.240) |
| College and above | -0.153 | -0.266* | -0.353** | -1.019*** |
|  | (0.138) | (0.116) | (0.129) | (0.301) |
| Marital status (married or cohabit) | -0.310*** | -0.158*** | -0.113** | 0.027 |
|  | (0.042) | (0.031) | (0.041) | (0.098) |
| Self-assessed health status (ref: poor) |  |  |  |  |
| Fair | -0.436*** | -0.476*** | -0.459*** | -0.389*** |
|  | (0.023) | (0.021) | (0.034) | (0.097) |
| Good | -0.820*** | -0.836*** | -0.771*** | -0.653*** |
|  | (0.028) | (0.028) | (0.048) | (0.109) |
| Whether are grandparents living with their children or not? (yes) | -0.043* | 0.001 | 0.031 | -0.129 |
|  | (0.020) | (0.020) | (0.035) | (0.099) |
| Number of grandchildren | 0.018** | 0.000 | -0.008 | 0.002 |
|  | (0.006) | (0.006) | (0.007) | (0.022) |
| Number of children | 0.014 | 0.037*** | 0.004 | 0.015 |
|  | (0.012) | (0.010) | (0.011) | (0.027) |
| Per capita household expenditure (ref: 1st quartile) | |  |  |  |
| 2nd quartile | -0.091*** | -0.066** | -0.028 | 0.042 |
|  | (0.025) | (0.024) | (0.040) | (0.118) |
| 3rd quartile | -0.121*** | -0.092*** | -0.098* | 0.005 |
|  | (0.026) | (0.025) | (0.042) | (0.108) |
| 4th quartile | -0.130*** | -0.064* | -0.175*** | -0.024 |
|  | (0.028) | (0.029) | (0.049) | (0.148) |

Note: † p < 0.1, * p<0.05, ** p<0.01, *** p<0.001.
